# Adrenal oncocytoma masquerading as a functional tumor

**DOI:** 10.4103/0970-1591.30275

**Published:** 2007

**Authors:** Tanmaya Goel, Joseph Thomas, Shveta Garg, Anuradha C. K. Rao, Sreedhar Reddy

**Affiliations:** Department of Urology, Kasturba Medical College (KMC), Manipal, India; * Department of Pathology, Kasturba Medical College (KMC), Manipal, India

**Keywords:** Adrenal oncocytoma, catecholamines, adrenalectomy, immunostaining

## Abstract

Adrenal oncocytoma is a rare entity, with 20 cases reported in literature. A functional oncocytoma is extremely rare. We present a case of adrenal oncocytoma in a hypertensive male who had elevated catecholamine levels, which improved after adrenalectomy with decrease in daily antihypertensive requirement.

## INTRODUCTION

Adrenal oncocytoma is a rare entity, with around 20 cases reported in world literature after surgical extirpation. Though it is a benign and nonfunctional tumor, rarely, we come across a functional adrenocortical neoplasm. We present an adrenal oncocytoma which mimicked a functional adrenal tumor.

## CASE REPORT

A 48-year-old male, a known diabetic and hypertensive for the last six years underwent a routine health checkup. Ultrasonography abdomen revealed a well-circumscribed, hypoechoic left suprarenal mass lesion measuring approximately 5 × 4.8 cm. His diabetes was well controlled, but the blood pressure (BP) was around 168/94 mmHg on regular treatment with three drugs (Tab. Atenolol 50 mg OD, Tab. Amlodipine 5 mg OD, Tab. Clonidine 150 micro gm BD). Though occasional palpitations were present, there was no history of headache, seizures, flushing, diaphoresis, weakness of limbs, abdominal striae, petechie or cushingoid features. Abdominal examination did not reveal any palpable mass, striae or bruit. All examinations, cardiac, ophthalmic, neurological were within normal limits.

**Figure 1:**
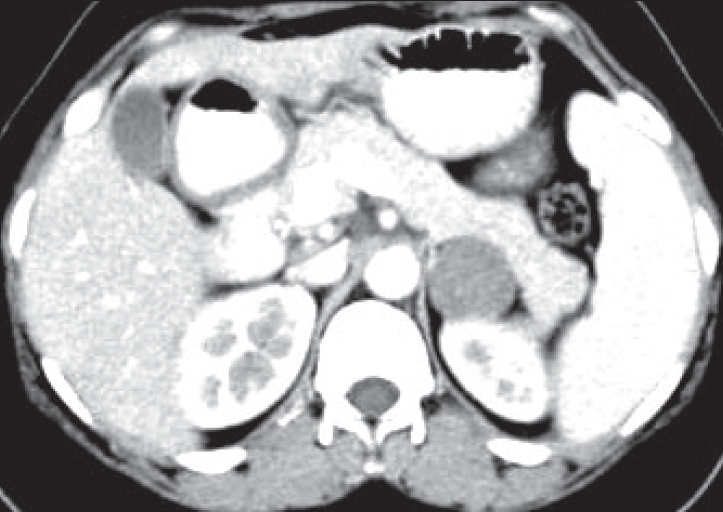
CECT abdomen depicting a well circumscribed, encapsulated non enhancing left adrenal mass lesion

The 24h urine estimation of Vinyl mandellic acid was 7.3 mg/vol (normal 2 to 10) and catecholamines were 236 microgm/vol (normal 0 to 100). Contrast enhanced computerized tomography (CECT) abdomen revealed a well-circumscribed nonenhancing 5.3 × 4.9 cm left suprarenal mass lesion [Figure 1]. In view of high blood pressures, mass characteristics and doubtful functional status, the patient underwent left adrenalectomy under coverage of alpha blockers. Intraoperatively, there were no marked fluctuations in the blood pressure and the surgery proceeded uneventfully.

### Pathological findings

Grossly, the specimen weighed 40g and measured 5.2 × 5.0 × 3 cm. The external surface was smooth with an intact capsule. Cut section showed yellow to brown areas. There was no evidence of hemorrhage or necrosis. Normal adrenal gland was identified compressed at the periphery. Multiple sections were taken from different areas for histopathological examination.

**Figure 2:**
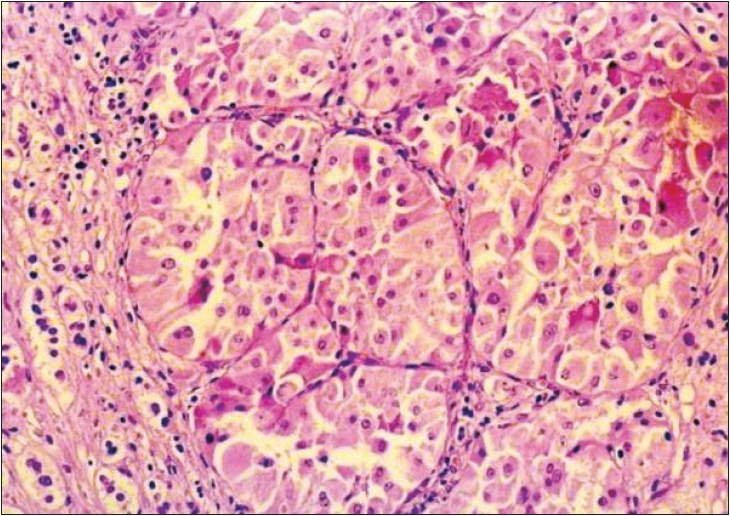
Photomicrograph showing dense eosinophilic oncocytes with normal adrenal tissue at periphery (H/E, 40×)

Microscopically, the tumor showed nests and diffuse sheets of polygonal cells with abundant granular eosinophilic cytoplasm, pleomorphic nuclei with central nucleoli. Few multinucleate giant cells were also evident. The nests of cells were surrounded by capillary network. There was no evidence of mitosis, necrosis, capsular or vascular invasion. Normal adrenal tissue was seen at the periphery [Figure 2]. The tumor cells were negative for Grimelius argyrophilic stain and did not show immunoreactivity for chromogranin A, NSE and S100. Immunostaining of tumor cells was positive for vimentin and cytokeratin; all diagnostic of an adrenal oncocytoma.

The postoperative period was uneventful and the daily quantum of antihypertensives required reduced significantly. His BP could be controlled only with Tab. Atenolol 25 mg OD. After six months, repeat CECT abdomen was normal. The BP was well under control (average 146/ 84 mm Hg), with reduction in 24h urine excretion of catecholamines to 64 microgm/vol. The patient is on regular follow-up.

## DISCUSSION

Neoplasms composed entirely of oncocytes are well described in the kidney, thyroid, salivary glands and other rare sites like the pituitary, parathyroid, lacrimal gland, respiratory tract and choroid plexus.[1] Oncocytic adrenal neoplasms are very rare with 20 cases reported in world literature. All these reported cases were nonfunctional and found incidentally at autopsy or on radiological investigations.[2,3] Only a single case of functional adrenal oncocytoma is reported where the patient had Cushing's syndrome.[4]

Adrenal oncocytomas usually occur in the age group of 27-72 years with more cases documented in females. These tumors vary in size from 3-15 cm and weigh between 3-865g. Fibrous encapsulation is characteristically seen in all these tumors and on cut section tend to be mahogany to tan brown. Microscopically, these tumor cells are arranged in nests, solid, trabecular, papillary or tubular patterns. On electron microscopy, tumor cells show densely packed mitochondria.[5] Whether the tumor is benign or malignant, can be predicted by its histological findings.[6] Adrenal oncocytoma can be differentiated grossly from renal oncocytomas by lack of central scar and lack of hemorrhagic areas on cut section. Since the cells have abundant granular cytoplasm, they have to be differentiated from pheochromocytomas. In our case, since the patient was hypertensive with elevated catecholamine levels on workup, this differential was considered and ruled out on the basis of morphology, cytochemistry and immunohistochemistry.[7] The settling of blood pressure (with decreased daily antihypertensive requirement) along with normalization of the catecholamines levels after surgical excision pointed to an adrenal oncocytoma masquerading as a functional tumor.
